# Label-Free Cancer Detection: From Intrinsic Biological Signatures to Intelligent Diagnostic Platforms

**DOI:** 10.3390/bioengineering13101119

**Published:** 2026-09-25

**Authors:** Raphaël F. Canadas

**Affiliations:** RISE-Health, Faculty of Medicine, University of Porto, Alameda Prof. Hernâni Monteiro, 4200-319 Porto, Portugal; rfcanadas@med.up.pt

Cancer continues to impose a major global health burden, with an estimated 20.6 million new cases and 9.8 million cancer-related deaths worldwide in 2024 [1]. Earlier and more accurate detection remains one of the most effective opportunities to improve patient outcomes, yet many established diagnostic approaches depend on invasive sampling, exogenous contrast agents or molecular labels, lengthy sample preparation, and centralized laboratory infrastructure. Label-free approaches seek to overcome some of these limitations by extracting diagnostically relevant information directly from intrinsic physical, chemical, mechanical, electrical, morphological, or imaging properties of cells, tissues, and biological fluids. Such approaches include optical and spectroscopic measurements, electrical impedance, cellular mechanics and morphology, molecular mass profiles, and quantitative imaging. Combined with advances in microfluidics, biosensing, high-resolution imaging, and artificial intelligence (AI), these methods are creating new opportunities for rapid, minimally invasive, and potentially real-time cancer assessment. Importantly, label-free does not imply information-poor: instead, these platforms can generate complex multidimensional datasets whose diagnostic value increasingly depends on robust computational analysis. The Special Issue “Label-Free Cancer Detection” brings together five contributions, three original research articles and two reviews, that illustrate both the breadth of this field and the challenges that still limit its translation into routine cancer care.

A central theme of this Special Issue is the use of intrinsic biophysical and molecular signatures to characterize malignancy with minimal sample perturbation. Calejo et al. [2] review label-free cancer detection based on biophysical cell phenotypes, covering phase-contrast and holographic microscopy, flow-based and microfluidic approaches, dynamic light scattering, atomic force microscopy, and electrical impedance spectroscopy. These methods interrogate features such as cell size, morphology, deformability, mechanics, and electrical behavior, offering information that can complement conventional molecular biomarkers and support liquid-biopsy applications. Vera et al. [3] focus this perspective on non-muscle invasive bladder cancer, where urine provides an accessible source of tumor-derived material. In their review, they discuss Raman and surface-enhanced Raman spectroscopy, interferometric flow cytometry, and electrochemical biosensing, while emphasizing recurrent translational barriers, including variability in sample handling, small clinical cohorts, limited multicenter validation, and insufficient evidence regarding cost-effectiveness and real-world diagnostic performance. Together, these reviews show the considerable technological diversity of label-free detection, but also reinforce the need for standardized workflows, optimized processing of liquid biopsies, harmonized acquisition and processing procedures, and validation in clinically representative populations.

The research articles further illustrate how label-free information can be coupled to computational analysis and clinically relevant imaging. Zhang et al. [4] combined serum mass-spectrometry proteomic profiles with eight machine-learning algorithms for cervical cancer detection, showing how multidimensional patterns may provide information beyond individual candidate biomarkers and highlighting the growing importance of interpretable AI. Iacob et al. [5] used multiparametric magnetic resonance imaging to evaluate periprostatic and subcutaneous adipose-tissue measurements as non-invasive indicators of prostate cancer risk. Their work is equally informative, demonstrating that technically measurable label-free features do not necessarily translate into robust clinical biomarkers and that more sophisticated quantitative phenotyping may be required. Yan et al. [6] broaden the concept beyond primary diagnosis by presenting a marker-less approach for real-time lung tumor tracking during image-guided radiotherapy. Their cross-patient deep-learning framework achieved a median three-dimensional tumor center tracking error of 1.53 mm in a feasibility study, illustrating how the avoidance of artificial markers may also benefit treatment guidance. Collectively, these studies underline both the promise of data-rich label-free measurements and the necessity of rigorous external validation before high performance in development cohorts can be interpreted as clinical utility. They also emphasize that clinically useful systems must be assessed not only for analytical performance but also for reproducibility, robustness, interpretability, and added value relative to current standards of care.

Taken together, the contributions to this Special Issue show that label-free cancer detection is progressing from individual sensing modalities toward increasingly integrated approaches in which engineering, cancer biology, clinical medicine, and data science converge. Developments outside this collection reinforce the same direction: label-free microscopy and vibrational spectroscopy are being investigated for rapid tissue assessment and intraoperative tumor identification [7,8], while recent work has combined phase holotomographic microscopy with pretrained neural networks to identify tumor-cell phenotypes associated with tumor grade and response to anticancer treatment [9]. At the same time, the studies gathered here make clear that important limitations remain. Pre-analytical variability, device and instrument calibration, methodological standardization, model interpretability, dataset shift, and integration into clinical workflows all require further attention. Future research should therefore prioritize prospective multicenter validation, appropriate benign and disease-control populations, transparent and reproducible AI pipelines, and multimodal strategies that combine complementary intrinsic signals. Particular attention should also be given to an optimal collection and processing of liquid biopsies for non-invasive cancer detection, and early-stage disease, where biological signals may be weaker and tumor heterogeneity more difficult to capture. Translation will further depend on practical considerations such as measurement speed, automation, robustness, cost, required expertise, patient acceptability, and compatibility with existing diagnostic pathways.

In conclusion, this Special Issue captures a field that has already achieved substantial technological and computational progress while remaining at an important translational stage. The five contributions demonstrate how intrinsic biological and imaging signatures can support minimally invasive detection, phenotyping, and treatment guidance, while also revealing where current approaches remain insufficient. The next challenge is not simply to generate increasingly sensitive signals but to establish that these signals are reproducible, interpretable, and clinically actionable across diverse patient populations and real-world settings. Continued integration of sensing technologies with quantitative imaging and explainable AI may enable more scalable approaches to cancer screening, diagnosis, monitoring, and treatment guidance. Achieving this will require closer collaboration among engineers, biologists, clinicians, and data scientists, as well as study designs that consider implementation early enough for promising proof-of-concept systems to move efficiently into prospective clinical evaluation. I would like to thank all authors, reviewers, and members of the editorial team who contributed to this Special Issue and hope that this collection will stimulate further interdisciplinary work toward robust, accessible, and clinically meaningful label-free cancer technologies.
